# Molecular Detection of Tick-Borne Agents in Cats from Southeastern and Northern Brazil

**DOI:** 10.3390/pathogens11010106

**Published:** 2022-01-16

**Authors:** Marcos Rogério André, Ana Cláudia Calchi, Maria Eduarda Chiaradia Furquim, Isabela de Andrade, Paulo Vitor Cadina Arantes, Lara Cristina de Melo Lopes, Iuri Kauan Lins do Nascimento Demarchi, Mayra Araguaia Pereira Figueiredo, Cirilo Antonio de Paula Lima, Rosangela Zacarias Machado

**Affiliations:** 1 Laboratório de Imunoparasitologia, Departamento de Patologia, Reprodução e Saúde Única—Faculdade de Ciências Agrárias e Veterinárias, Universidade Estadual Paulista, UNESP, Jaboticabal 14884-900, SP, Brazil; ana.calchi@unesp.br (A.C.C.); mecfurquim@yahoo.com.br (M.E.C.F.); isaandrade5743@gmail.com (I.d.A.); paulocadina@gmail.com (P.V.C.A.); laracristinalopes@hotmail.com (L.C.d.M.L.); rzacariasmachado@gmail.com (R.Z.M.); 2 Laboratório de Parasitologia, Entomologia e Biologia Molecular Aplicada à Saúde Única, Universidade Federal de Rondônia, Rolim de Moura 76940-000, RO, Brazil; iuri.demarchi98@gmail.com (I.K.L.d.N.D.); mayra.araguaia@unir.br (M.A.P.F.); 3 Departamento de Cirurgia Veterinária, Universidade Federal de Uberlândia, Uberlândia 38400-902, MG, Brazil; paulalima@ufu.br

**Keywords:** feline, *Anaplasma*, *Ehrlichia*, *Babesia*, *Cytauxzoon*, *Hepatozoon*

## Abstract

Even though the epidemiology of tick-borne agents (TBA) in dogs has been extensively investigated around the world, the occurrence, vectors involved, and molecular identity of these agents in cats remains elusive in many regions. Among TBA, *Ehrlichia*, *Anaplasma*, *Babesia*, *Cytauxzoon*, and *Hepatozoon* are responsible for diseases with non-specific clinical signs in cats, making essential the use of molecular techniques for accurate diagnosis and proper treatment. The present work aimed to investigate the occurrence and molecular identity of tick-borne agents (*Ehrlichia*, *Anaplasma*, *Babesia*/*Theileria*, *Cytauxzoon*, and *Hepatozoon*) in cats from southeastern (states of São Paulo (SP) and Minas Gerais (MG)) and northern (state of Rondônia (RO)) Brazil. For this purpose, 390 blood samples were collected from domiciled cats in MG (*n* = 155), SP (*n* = 151), and RO(*n* = 84) states, submitted to DNA extraction and PCR assays for *Ehrlichia* spp. (*dsb* gene), *Anaplasma* spp. (*rrs* gene), piroplasmids (18S rRNA gene), and *Hepatozoon* spp. (18S rRNA gene), sequencing, and phylogenetic inferences. The overall positivity for *Anaplasma* spp., *Ehrlichia* spp., *Babesia/Theileria* spp., *Cytauxzoon* spp., and *Hepatozoon* spp. were 7.4% (12.3% (MG) and 6.6% (SP)), 2% (4.5% (MG) and 0.6% (SP)), 0.7% (0.6% (MG), 0.6% (SP) and 1.2% (RO)), 27.2% (41.9% (MG), 24.5% (SP) and 4.8% (RO), and 0%, respectively. The phylogenetic analysis grouped the obtained sequences with ‘*Candidatus* Anaplasma amazonensis’, *A. platys*, *B. vogeli*, and *Cytauxzoon* sp. previously detected in wild felids from Brazil. qPCR specific for *E. canis* based on the *dsb* gene confirmed the molecular identity of the detected ehrlichial agent. The present study expanded the list and geographical distribution of hemoparasites in cats. ‘*Candidatus* Anaplasma amazonensis’, recently detected in sloths from northern Brazil, was described for the first time in cats. This is the first report of piroplasmids infecting cats in northern Brazil. Coinfection by *Cytauxzoon* and other TBA (*Ehrlichia, Anaplasma*, and *B. vogeli*) reported in the present study raises the need for veterinary practitioners’ awareness of cats parasitized by multiple TBA.

## 1. Introduction

Even though the epidemiology of tick-borne agents (TBA) in dogs has been extensively investigated around the world, the occurrence, vectors involved, and molecular identity of these agents in cats remains elusive in many regions. Among TBA, *Ehrlichia*, *Anaplasma*, *Babesia*, *Cytauxzoon*, and *Hepatozoon* are responsible for diseases with non-specific clinical signs in cats, making essential the use of molecular techniques for accurate diagnosis and proper treatment.

*Ehrlichia* spp. and *Anaplasma* spp. (Rickettsiales: Anaplasmataceae) comprise Gram-negative obligate intracellular α-Proteobacteria that infect erythrocytes, leucocytes, and platelets of several vertebrate species and are mainly transmitted by tick bites [1]. Feline ehrlichiosis and anaplasmosis are characterized by unspecific clinical signs and laboratorial abnormalities, such as anorexia, lethargy, fever, dehydration, anemia, thrombocytopenia, leucocytosis/leucopenia, and pancytopenia [2,3]. The geographical occurrence of *Anaplasma* and *Ehrlichia* is associated with that found for their competent vectors: while *R. sanguineus* sensu lato and sensu stricto are the main vectors for *E. canis* [4] and *A. platys* [5], respectively, *Ixodes pacificus/I. scapularis* and *Ixodes ricinus* are responsible for *A. phagocytophilum* transmission in the USA and Europe, respectively [3].

Feline piroplasmids (Piroplasmida: Babesiidae/Theileriidae) comprise *Babesia* spp. [6], *Theileria* spp. [7,8], and *Cytauxzoon* spp. [9]. While clinical babesiosis associated with infection by *Babesia felis*, *B. leo*, *B. lengau*, and “*Babesia* species cat Western Cape” has been reported mainly in cats from South Africa, clinical cytauxzoonoosis due *Cytauxzoon felis* has been reported mostly in cats from the USA [9]. Feline babesiosis is mainly characterized by lethargy, anorexia, and anemia, whereas *B. lengau* caused cerebral babesiosis [6]. Macrophages infected by *Cytauxzoon felis*-schizonts can disseminate in several tissues, causing thrombosis, circulatory impairment, systemic inflammatory response, and eventually death. On the other hand, piroplasms parasitizing red blood cells lead to hemolytic anemia and erythrophagocytosis [10]. In the USA, cytauxzoonosis is characterized by a (per)acute severe febrile disease, with non-specific clinical and hematological signs (anorexia, fever, dyspnea, tachycardia, generalized pain, vocalization, anemia, thrombocytopenia, leucopenia, hepato/splenomegaly, neurological signs, etc.) [10]. Even though piroplasmids phylogenetically related to *Theileria equi* and ruminant-associated *Theileria* have been detected in cats from Brazil [7,8], clinical signs associated with infection by these theileriids have not been reported. *Amblyomma americanum* and *Dermacentor variabilis* are the main vectors of *Cytauxzoon felis* in the USA [10], whereas the vectors for feline-associated *Babesia* are still unknown.

Apicomplexan protozoans of the genus *Hepatozoon* sp. (Adeleorina: Hepatozoidae) share a general life cycle, which includes sexual reproduction and sporogony in a hematophagous invertebrate definitive host and merogony followed by gametogony in an intermediate vertebrate host. *Hepatozoon* transmission occurs by ingestion of the definitive host, represented by an invertebrate containing *Hepatozoon* oocysts, by the intermediate host [11]. Although the transmission route has not yet been elucidated for feline hepatozoonosis, the association of *Hepatozoon* infection in cats with outdoor access suggests the potential transmission by arthropod vectors or by predation [12]. Indeed, *Hepatozoon felis* DNA was detected in *Rhipicephalus sanguineus* sensu lato ticks in Turkey [13] and Portugal [14]. Even though feline hepatozoonosis caused by *H. felis* is usually subclinical and the majority of infected cats presented no specific clinical signs [12], a fatal infection caused by *Hepatozoon silvestris* with the presence of *Hepatozoon* meronts associated with lymphoplasmacytic and histiocytic myocarditis was reported in a cat from Switzerland [15].

Considering that several vector-borne agents may occur in cats, the present work aimed to investigate the occurrence and molecular identity of tick-borne agents (*Ehrlichia*, *Anaplasma*, *Babesia*/*Theileria*, *Cytauxzoon*, and *Hepatozoon*) in cats in southeastern (states of São Paulo and Minas Gerais) and northern (state of Rondônia) Brazil.

## 2. Results

### 2.1. PCR Assays for Gapdh mammalian Endogenous gene

All cats’ blood samples were positive in the PCR assay for the endogenous gene (*gapdh*). The mean genomic DNA concentration, 260/280, and 260/230 ratios were 35.37 ng/µL, 1.77, and 0.57, respectively.

### 2.2. PCR Assays for Anaplasma spp. and Ehrlichia spp.

Out of the 390 samples analyzed, 7.4% (12.3% [19/155] and 6.6% [10/151] cats from Minas Gerais and São Paulo state, respectively) were positive in the nPCR based on the 16S rRNA gene of *Anaplasma* spp. Out of the 26 positive samples, 5 were chosen for sequencing due to the higher band intensity observed in the agarose gel electrophoresis. All 26 samples were negative in PCR assays based on the *gltA* gene and the 23S-5S intergenic region.

A total of 2% out of 390 cats (4.5% [7/155] and 0.6% [1/151] of cats from the states of Minas Gerais and São Paulo, respectively) were positive in the cPCR assay for *Ehrlichia* spp. based on the *dsb* gene. Unfortunately, no samples were sequenced due to low band intensity on agarose gel electrophoresis. In front of that, a qPCR assay specific for *E. canis* and also based on the *dsb* gene was performed on these positive samples. As a result, three samples (37.5% [3/8]) were positive in the qPCR, but the samples were not quantified due to the low amount of *E. canis* DNA in the tested samples (Monte Carlo effect). The efficiency, R^2^, slope, and Y-intercept showed values of 99.7%, 0.988, −3.330, and 40.629, respectively. No samples were positive in PCR assays based on *groEL, sodB,* and *omp-1* genes, precluding additional molecular characterization.

### 2.3. PCR Assays for Piroplasmida

#### 2.3.1. *Babesia* spp. and *Theileria* spp.

Out of the 390 samples analyzed, 0.7% (0.6% [1/155] from Minas Gerais, 0.6% [1/151] from São Paulo, and 1.2% [1/84]) were positive in the nPCR based on the 18S rRNA gene of *Babesia* spp./*Theileria* spp. No samples were positive in the PCR assays for additional molecular characterization based on the *cox-1*, *hsp70*, *β-tubulin*, *cytB*, and the intergenic region (ITS1).

#### 2.3.2. *Cytauxzoon* spp.

Out of the 390 samples, 27.2% (41.9% [65/155], 24.5% [37/151], and 4.8% [4/84] from Minas Gerais, São Paulo, and Rondônia states, respectively) were positive in the PCR based on the 18S rRNA gene of *Cytauxzoon* spp. Out of the 106 positive samples, seven were chosen for sequencing due to higher band intensity in the agarose gel electrophoresis. No samples were positive in the nPCR assay based on the intergenic region (ITS-1).

#### 2.3.3. *Hepatozoon* spp.

No blood sample was positive for *Hepatozoon* spp. in the nPCR based on the 18S rRNA gene.

### 2.4. Co-positivity for Anaplasmataceae and Piroplasmida Agents

In total, 10 samples showed co-positivity between the tested agents. Out of these, eight were positive for both *Cytauxzoon* sp. and *Anaplasma* sp. (six and two from Minas Gerais and São Paulo, respectively), one for *Cytauxzoon* sp. and *Ehrlichia* sp., and one for *Cytauxzoon* sp. and *B. vogeli*, both from the state of Minas Gerais (Table 1).

### 2.5. BLAST and Phylogenetic Analyses

BLASTn analysis results are presented in Table 2.

**Figure 1:**
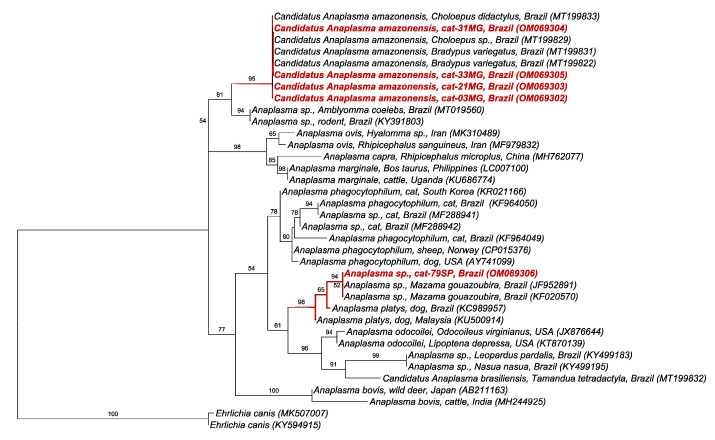
Phylogenetic tree based on an alignment of 246 bp of *Anaplasma* sp. *rrs* gene, using maximum likelihood method and TPM2 + I + G as an evolutionary model. Sequences from the present study were highlighted in red. *Ehrlichia canis* was used as an outgroup.

The phylogenetic analysis by ML and TPM2 + I + G evolutionary model (246 bp alignment) based on the *rrs* gene of *Anaplasma* spp. positioned the sequences detected in cats from Minas Gerais state into the ‘*Candidatus* Anaplasma amazonensis’ clade, recently described in sloths sampled in Brazil with 95% branch support. On the other hand, the sequence detected in a cat from São Paulo state was allocated with sequences of *Anaplasma* sp. detected in gray-brocket deer (*Mazama gouazoubira*) from Brazil, with 94% branch support in a clade close to *Anaplasma platys* clade (Figure 1).

**Figure 2:**
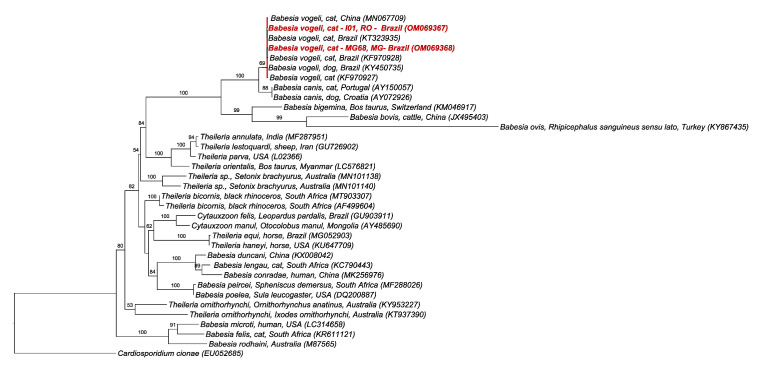
Phylogenetic tree based on an alignment of 605 bp of piroplasmids 18S rRNA gene, using maximum likelihood method and TIM3 + I + G as an evolutionary model. Sequences from the present study were highlighted in red. *Cardiosporidium cionae* was used as an outgroup.

The phylogenetic analysis by ML and TIM3 + I + G evolutionary model (605 bp alignment) based on the 18S rRNA gene of piroplasmids positioned the sequences detected in one cat from Minas Gerais and one cat from Rondônia into the *Babesia vogeli* clade previously detected in dogs and cats from Brazil and China, with 69% branch support. Unfortunately, the sequence detected in one cat from São Paulo state presented low quality and was removed from the analysis (Figure 2).

**Figure 3:**
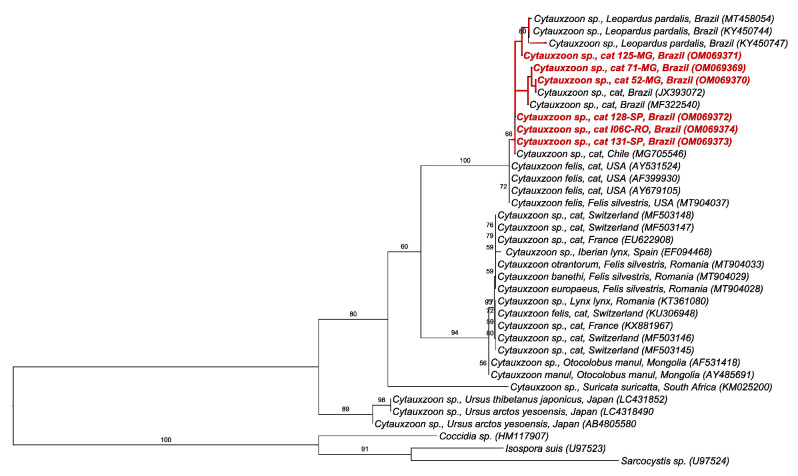
Phylogenetic tree based on an alignment of 118 bp of *Cytauxzoon* sp. 18S rRNA gene, using maximum likelihood method and TPM3 + G as an evolutionary model. Sequences from the present study were highlighted in red. *Coccidia* sp., *Isospora suis*, and *Sarcocystis* sp. were used as an outgroup.

Finally, the phylogenetic analysis by ML and TPM3 + G evolutionary model (118 bp alignment) based on the 18S rRNA gene of *Cytauxzoon* spp. positioned the sequences detected in cats from this study in the same clade containing *Cytauxzoon* sp. sequences detected in cats and ocelots from Brazil and Chile, with 66% branch support (Figure 3).

## 3. Discussion

Herein, the positivity in the nPCR assay for *Anaplasma* sp. was 7.4% (12.3% (MG) and 6.6% (SP)). Previous studies conducted in Brazil reported molecular positivity ranging from 3.7% to 8% [7,16,17]. The present study showed, for the first time, the occurrence of ‘*Candidatus* Anaplasma amazonensis’ in cats and the first molecular detection of *Anaplasma* sp. in cats in the state of Minas Gerais, adding to the list of *Anaplasma* species/genotypes that can infect cats in Brazil. ‘*Candidatus* Anaplasma amazonensis’ has been recently detected in sloths from northern Brazil [18]. Additionally, a genotype closely related to *Anaplasma* previously detected in brocket deer (*Mazama gouazoubira*) and dog-associated *A. platys* were also detected in cats in the present study. The pathogenic potential of these *Anaplasma* genotypes in cats is still unknown. For instance, even though *A. platys* was molecularly detected in a cats presenting apathy, anorexia, thrombocytopenia, and leucocytosis in the state of Pernambuco, northeastern Brazil, the PCR-positivity for *A. platys* was not associated with clinical signs and hematological abnormalities in cats sampled in the state of Rio de Janeiro [17,19]. *Anaplasma rrs* genotypes closely related to *A. phagocytophilum* have been previously detected in cats in the states of São Paulo [7], Rio Grande do Norte [20], and Santa Catarina [16]. Considering that competent tick vectors (*Ixodes persulcatus* complex) for *A. phagocytophilum* are not present in Brazil, these *rrs* genotypes might represent a putative novel species yet to be isolated and better characterized. On the other hand, *Anaplasma phagocytophilum* has been the main Anaplasmataceae agent detected in cats in the USA and Europe, where the molecular occurrence ranged from 0% to 6.9% and 0% to 23.1% (reviewed by Schafer and Kohn, 2020 [3]). Unfortunately, the lack of amplification for three additional molecular markers (*gltA, groEL,* and ITS 23S-5S) precluded further molecular characterization. Considering that we only obtained a small fragment of the *rrs* gene, future phylogenetic inferences based on larger fragments are needed in order to confirm the genetic identity of these *Anaplasma* genotypes detected in cats.

The positivity in the PCR assay for *E. canis* in cats found in the present study was 2% (MG 4.5%; SP 0.6%; RO 0%), whereas previous studies from Brazil reported positivity rates ranging from 1% to 20% [8,16,17,21,22]. Indeed, *E. canis* has been shown to be the most frequently detected Anaplasmataceae agent in cats from Brazil, and it has already been reported in the states of Minas Gerais [23], Maranhão [21], Mato Grosso [22], Mato Grosso do Sul [8], Rio Grande do Norte [20], Rio de Janeiro [17], and Santa Catarina [16]. Additionally, *E. canis* has already been detected in cats from the USA [24], Portugal [14], and Angola [25]. Even though the low intensity of the obtained *dsb* amplicons in the agarose gel electrophoresis precluded the sequencing of the amplified products in the conventional PCR for *Ehrlichia* spp., a qPCR assay specific for *E. canis* based on the *dsb* gene confirmed the molecular identity of the ehrlichial agent infecting some cats in the states of São Paulo and Minas Gerais. Among the eight samples positive in the cPCR for *Ehrlichia* spp., three also showed to be positive in the qPCR for *E. canis*. Even though qPCR is considered to be more sensitive than cPCR, the Monte Carlo effect [26] might have hampered the detection and reproducibility in samples with a very low amount of *E. canis* DNA. Considering that both *E. canis* and its vector, the brown dog tick (*R. sanguineus* sensu lato) are endemic in most of the Brazilian states, except for the state of Rio Grande do Sul [5,27], the occurrence of *E. canis* in cats in different Brazilian regions might reflect the prevalence of both pathogen and tick vector in dogs and the environmental infestation with *R. sanguineus* sensu lato. The pathogenic potential of *E. canis* in cats, solely or associated with other hemoparasites, deserves further studies. Anemia was statistically associated with seropositivity to *E. canis* in cats from Rio de Janeiro [17]. A cat positive in the PCR for *E. canis* in the state of Minas Gerais showed to be thrombocytopenic [23]. *Ehrlichia canis*-PCR-positive cats in the state of Mato Grosso, central-western Brazil, showed a tendency to be lymphopenic and thrombocytopenic [22]. This is the first report on the occurrence of *E. canis* in cats from the state of São Paulo.

A low positivity (0.7%) for *Babesia*/*Theileria* (MG 0.6%; SP 0.6%; RO 1.2%) was found among cats sampled in the present study. This is the first report of *B. vogeli* in cats from the states of Rondônia e Minas Gerais. Previous studies in Brazil found positivity rates ranging from 4% to 16% in cats in the states of São Paulo [7], Mato Grosso do Sul [8], and Rio Grande do Sul [27]. *Babesia vogeli* has also been detected in cats from Thailand [28], Portugal [29], Saint Kitts [30], and Qatar [31]. According to Penzhorn (2020) [6], the widespread distribution of dog-associated *B. vogeli* in cats is not a surprise due to the cosmopolitan distribution of its vector, the tick *R. sanguineus* sensu lato. It seems that immunocompetent cats deal with the infection without discernible clinical and hematological abnormalities. Nevertheless, experimental studies should be performed in order to check the progression of *B. vogeli* infection in cats.

On the other hand, high positivity in the PCR for *Cytauxzoon* was found among cats sampled in the states of Minas Gerais (41.9%) and São Paulo (24.5%). This is the first report of *Cytauxzoon* in domestic cats in the three mentioned states. Cats sampled in the states of SP and MG, where higher numbers of PCR-positive cats were observed, were apparently healthy since they were submitted to spay/neutering procedures. Apparently, these cats might have been infected by a non-pathogenic strain of *Cytauxzoon*. This high number of positive cats might be related to exposure to an infected tick vector endemic to both areas. Unfortunately, information about outdoor access and exposure to ticks were not available, precluding the assessment of associated risk factors for positivity. Some cats sampled in SP and MG were parasitized by *Ctenocephalides felis felis* fleas at the time of blood sampling (data not shown) [32]. It is most likely that positive cats might have presented long-lasting erythroparasitemia and may act as reservoirs for non-pathogenic strains of *Cytauxzoon* in Brazil, which should be confirmed by experimental infection of cats with Brazilian strains of *Cytauxzoon*. Neotropical wild felids likely play a role as reservoirs for *Cytauxzoon* in Brazil [33,34], probably presenting short and self-limiting schizogony as previously observed in bobcats (*Lynx rufus*), which act as reservoirs for *C. felis* in the USA [9]. As far as authors are concerned, fatal cytauxzoonosis has not been reported in domestic cats in Brazil, despite reports of fatal cytauxzoonosis in lions (*Panthera leo*) [35] and jaguars (*Panthera onca*) [36] in the country. Actually, despite the detection of piroplasms in cats’ erythrocytes [20,37], schizonts have not been detected in domestic cats from Brazil so far. These findings suggest that several isolates may occur in the country, whose pathogenicity may differ from each other. The recent description of three new *Cytauxzoon* species, namely *C. otrantorum*, *C. banethi*, and *C. europaeus,* in wild felids from Europe expands the diversity of *Cytauxzoon* species other than *C. felis* and *C. manul* [38]. Herein, the 18S rRNA fragments of *Cytauxzoon* sp. detected in the sampled cats grouped with *Cytauxzoon* sequences previously detected in wild felids from Brazil and domestic cats from Chile. Even though the fragment used was conserved and small, the topology suggests a slight separation between *Cytauxzoon* isolates from Brazil and *C. felis* from the USA. Nonetheless, these findings should be confirmed with phylogenetic assessments based on near-full 18S rRNA and mitochondrial genes. The apparent low pathogenicity of Brazilian strains of *Cytauxzoon* differs from that observed for North American *C. felis* isolates, despite the phylogenetic proximity between them. Interestingly, European species of *Cytauxzoon* also seem to show low pathogenicity when compared to *C. felis*. In front of that, we suggest using the denomination *Cytauxzoon* sp. for Brazilian isolates instead of *C. felis* until further data obtained from mitochondrial and whole-genome sequencing are achieved, allowing a better phylogenetic/phylogenomics positioning.

None of the sampled cats showed to be positive in a nested PCR assay for *Hepatozoon* sp. based on the 18S rRNA. This finding was not a surprise since reports of *Hepatozoon* infection in cats in Brazil are scarce. The few works found in the literature reported a low molecular positivity (0.5%–1.6%) for *Hepatozoon* in cats in the states of Maranhão [39], Mato Grosso do Sul [8], and Mato Grosso [40]. In Brazil, phylogenetic studies based on the 18S rRNA showed that *H. canis*, *H. felis*, and *Hepatozoon*, closely related to *H. americanum,* can infect cats [8,39,40,41]. *Hepatozoon felis* has already been detected in cats from Spain [42], Italy [43,44], Angola [25], South Africa [45], Cabo Verde [46], and Austria [47]. On the other hand, *H. canis* has already been detected in cats from Spain [42] and Italy [43]. Following the same pattern for the previously mentioned tick-borne agents, the real significance of *Hepatozoon* infection in cats from Brazil is unknown. Previously, a cat naturally infected by *H. canis* in the state of São Paulo presented gamonts in neutrophils, renal failure, anorexia, lethargy, leucopenia, and severe anemia [41,48]. Clinical cases of feline hepatozoonosis without coinfection with other infectious agents were also described in cats infected by *Hepatozoon silvestris* and *H. felis* from Switzerland [15] and Austria [47], respectively.

Co-positivity by *Cytauxzoon* and one more TBA (*Anaplasma, Ehrlichia*, and *Babesia/Theileria*) was confirmed in 10 cats’ blood samples. Previously in Brazil, coinfection by *E. canis*, *Anaplasma* closely related to *A. phagocytophilum*, and *Cytauxzoon* was found in cats presenting non-specific clinical signs in the state of Rio Grande do Norte, northeastern Brazil [20]. Coinfection by several vector-borne agents in cats seems to be more usual than previously thought [8]. The consequences of coinfection by several TBA in cats deserve more attention by veterinary practitioners. Future studies aiming at verifying the impact of TBA in cats in single or co-infections are much needed.

Finally, keeping in mind that *A. platys* [49] and *E. canis* [48] have already been detected in humans and considering the close contact between cats and owners, special attention is needed regarding feline vector-borne agents. Regarding that, the role of cats as a sentinel for tick-borne diseases should not be neglected.

This body of work presents two main limitations. One of them relies on the fact that we were not able to sequence all the obtained amplicons due to the faint bands obtained in the agarose gel electrophoresis. Without sequencing, we could not confirm the molecular identity of the positive sample obtained in the nested PCR for *Babesia/Theileria* from a cat sampled in Jaboticabal, SP. Similarly, even though the used nested PCR assay for *Anaplasma* is considered specific for this genus, we cannot rule out the amplification of a closely related agent DNA using this protocol. Likewise, despite the few amplicons sequenced, the protocol used herein for detecting a fragment of *Cytauxzoon* 18S rRNA is considered quite specific for this Piroplasmida genus. Nonetheless, without sequencing all the amplicons, we cannot rule out the occurrence of false positives due to non-specific amplification. Because of that, we used the term “positivity in PCR assays” instead of occurrence throughout the manuscript. The second limitation is the lack of additional information regarding the clinical and laboratorial findings of the cats sampled in the present study, which precluded inferences on the possible association between the studied TBA and the manifestation of disease. In addition, the lack of information regarding epidemiological variables (e.g., outdoor access, history of ectoparasite infestation, contact with other animal species, estimated age, etc.) hampered the assessment of possible risk factors associated with the positivity for *Ehrlichia, Anaplasma*, and piroplasmids.

## 4. Materials and Methods

### 4.1. Cats’ Blood Sampling

**Figure 4:**
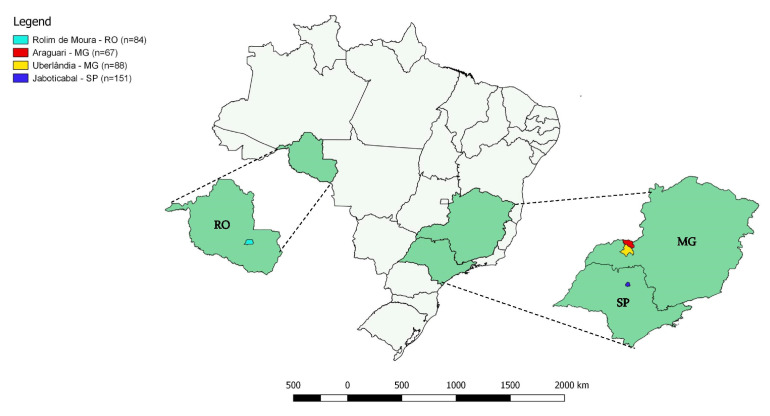
Map showing the location of the cities of Rolim de Moura (Rondônia State), Uberlândia and Araguari (Minas Gerais State), and Jaboticabal (São Paulo State), where cats were sampled in the present study.

Between the months of August and September 2018, 390 blood samples were collected from domiciled cats in Minas Gerais (155 samples—Uberlândia 19°00′39” S 48°05′45″ W and Araguari 18°24′43” S 49°03′09″ W), São Paulo (151 samples—Jaboticabal 21°15′33″ S 48°18′54″ W) and Rondônia (84 samples—Rolim de Moura 11°43′21.8″ S 61°46′29.5″ W) states (Figure 4). These samples were collected with the consent of the animals’ tutors during neutering campaigns promoted by the Animal Protector Association Neutering Center, located at the School of Agricultural and Veterinarian Sciences (FCAV/ UNESP, Jaboticabal, São Paulo State, Southeastern Brazil), Animal Populational Control Project a partnership by the Federal University of Uberlândia (UFU) and the city’s Zoonosis Control Center (Uberlândia, Minas Gerais State) [32] and by researchers of Universidade Federal de Rondônia. All procedures were authorized and approved by the Animal Use Ethics Committee of the Universidade Estadual Paulista (IACUC FCAV/UNESP 012017/17). Blood samples were collected in tubes containing EDTA, transported in liquid nitrogen to the laboratory, and stored in a freezer (−80 °C) until sample processing.

### 4.2. DNA Extraction from Cats’ Blood Samples and PCR Assays for Mammalian endogenous gene

DNA was extracted from 250 µL from each blood sample according to the protocol described by Kuramae-Izioka (1997) [50]. The quantification of total genomic DNA, as well as the measurement of the 260/280 and 230/280 ratios of the extracted desoxirrobonucleic acids, were performed in a Nanodrop device (Thermo Scientific^®^) by reading the absorbance of each sample. The presence of amplifiable DNA in cats’ blood samples was verified by a conventional PCR assay targeting the mammal endogenous glyceraldehyde-3-phosphate dehydrogenase (*gapdh*) gene [51]. Only the samples that were positive in this PCR assay were used in the following PCR assays aiming at detecting Anaplasmataceae and Piroplasmida DNA.

### 4.3. PCR Assays for Anaplasma spp. and Ehrlichia spp.

For *Anaplasma* spp. DNA detection, a nested (n)PCR assay based on the *rrs* gene was performed [52]. Subsequently, the positive samples for *Anaplasma* spp. were tested by an nPCR based on the *gltA* gene [53] and a conventional (c)PCR targeting the 23S-5S intergenic region of *Anaplasma* spp. [54], respectively (Table 3). For *Ehrlichia* spp. DNA detection, a cPCR based on the *dsb* gene was performed [55]. The positive samples for *Ehrlichia* spp. were tested by cPCR assays based on the *groEL* [56], *sodB* [57], and *omp-1* [58] genes (Table 3). The cPCR assays were performed using 5 μL of the DNA samples in a mixture containing 1.25 U Platinum Taq DNA Polymerase (Invitrogen, Carlsbad, California, USA), PCR buffer (PCR buffer 10×, 100 nM Tris-HCl, pH 9.0, 500 mM KCl), 0.2 mM deoxynucleotides (dATP, dTTP, dCTP, and dGTP) (Invitrogen, Carlsbad, California, United States), 1.5 mM of magnesium chloride (Invitrogen, Carlsbad, CA, USA), 0.5 μM of each primer (Invitrogen), and sterile ultrapure water (Invitrogen) q.s. 25 μL. In nPCR assays, 1 μL of the amplified product from the first PCR reaction was used as the target DNA in the second reaction. DNA samples from *A. phagocytophilum,* kindly provided by Professor John Stephen Dumler (Uniformed Services University of the Health Sciences, Bethesda, MD, USA), and *E. canis*, obtained from DH82 cells infected with the Jaboticabal strain of *E. canis* [59], were used as positive controls. Sterile ultrapure water (Nuclease-Free Water, Promega Corporation) was used as a negative control.

Additionally, a specific qPCR assay for *E. canis* based on the *dsb* gene [55] was performed on the positive samples for *Ehrlichia* sp. The assay was performed with a final volume of 10 μL containing 1 μL of DNA sample, 0.2 μM of each primer (F: 5′-TTGCAAAATGATGTCTGAAGATATGAAACA-3′ and R: 5′-GCTGCTCCACCAATAAATGTATCYCCTA-3′) and hydrolysis probe (5’ FAM AGCTAGTGCTGCTTGGGCAACTTTGAGTGAA-(BHQ-1-3′)), 5 μL GoTaq Probe qPCR Master Mix (Promega Corporation, Madison WI, USA), and sterilized ultrapure water (Nuclease-Free Water; Promega Corporation) q.s. 9 μL. The thermal conditions used were 95 °C for 5 minutes, followed by 40 cycles of 95 °C for 15 seconds and 60 °C for 1 minute. PCR amplifications were performed in low-profile multiplate unskirted PCR plates (Bio-Rad, Hercules, CA USA) using a CFX96 Thermal Cycler (Bio-Rad). Quantification of the number of copies of target DNA/μL was performed using IDT psmart plasmids (Integrated DNA Technologies, Coralville, IA, USA) containing the target sequences. Serial dilutions were performed to construct standard curves with different plasmid DNA concentrations (2.0 × 10^7^ to 2.0 × 10^0^ copies/μL). The number of plasmid copies/µL of the amount (g/µL) of DNA/plasmid (bp) was determined by multiplying by 6.022 × 10^23^. Each qPCR assay was performed in duplicate for each DNA sample. All duplicates showing cycle quantification (Cq) values differing by >0.5 were re-tested. Amplification efficiency (E) was calculated from the slope of the standard curve in each run (E = 10^−1/slope^). The reactions followed the standards established by the Minimum Information for Publication of Quantitative Real-time PCR Experiments [26].

### 4.4. PCR Assays for Piroplasmida

#### 4.4.1. *Babesia* spp. and *Theileria* spp.

An nPCR assay that amplifies a fragment of the 18S rRNA gene of piroplasmids (Jefferies et al., 2007) was performed to detect *Babesia/Theileria* spp. in the DNA samples. Positive samples in this assay were tested by cPCR assays based on the *cox-1* [61], *hsp70* [62], *β-tubulin* [63], and * cytB* [64] genes and the intergenic region (ITS-1) [65] (Table 3). The assays were performed using 5 μL of the DNA samples in a mixture containing 0.75 U Platinum Taq DNA Polymerase (Invitrogen, Carlsbad, CA, USA), PCR buffer (PCR buffer 10×—100 nM Tris-HCl, pH 9.0, 500 mM KCl), 0.2 mM deoxynucleotides (dATP, dTTP, dCTP, and dGTP) (Invitrogen, Carlsbad, CA, USA), 1.5 mM of Magnesium chloride (Invitrogen, Carlsbad, CA, USA), 0.5 μM of each primer (Invitrogen), and sterile ultrapure water (Invitrogen) q.s.25 μL. In nPCR assays, 1 μL of the amplified product from the first PCR reaction was used as the target DNA in the second reaction. A DNA sample obtained from a dog experimentally infected with *Babesia vogeli* (Jaboticabal strain) [70] was used as a positive control. Sterile ultrapure water (Nuclease-Free Water, Promega Corporation, Madison, WI, USA) was used as a negative control.

#### 4.4.2. *Cytauxzoon* spp.

PCR assays based on the 18S rRNA gene were performed to detect *Cytauxzoon* spp. DNA in cats’ blood samples [66]. Subsequently, the positive samples were subjected to an nPCR assay that amplified a fragment of the intergenic region (ITS-1) [65] (Table 3). The assays were performed using 5 μL of the DNA samples in a mixture containing 1.25 U Platinum Taq DNA Polymerase (Invitrogen, Carlsbad, California, USA), PCR buffer (PCR buffer 10×—100 nM Tris-HCl, pH 9.0, 500 mM KCl), 0.2 mM deoxynucleotides (dATP, dTTP, dCTP, and dGTP) (Invitrogen, Carlsbad, CA, USA), 1.5 mM of magnesium chloride (Invitrogen, Carlsbad, CA, USA), 0.4 μM of each primer (Invitrogen), and sterile ultrapure water (Invitrogen) q.s. 25 μL. In nPCR assays, 1 μL of the amplified product from the first PCR reaction was used as the target DNA in the second reaction. DNA samples of *Cytauxzoon* sp. from an ocelot sampled in the Pantanal of Mato Grosso do Sul [34] and ultrapure sterilized water were used as positive and negative controls, respectively.

### 4.5. PCR assay for Hepatozoon spp.

For detection of *Hepatozoon* spp. DNA, an nPCR assay based on the 18S rRNA gene was performed [67,68,69] (Table 3). The nPCR assay was performed using 5 μL of the DNA samples in a mixture containing 0.75 U Platinum Taq DNA Polymerase (Invitrogen, Carlsbad, CA, USA), PCR buffer (PCR buffer 10×—100 nM Tris-HCl, pH 9.0, 500 mM KCl), 0.2 mM deoxynucleotides (dATP, dTTP, dCTP, and dGTP) (Invitrogen, Carlsbad, CA, USA), 1.5 mM of magnesium chloride (Invitrogen, Carlsbad, CA, USA), 1.25 μM of each primer (Invitrogen), and sterile ultrapure water (Invitrogen) q.s.25 μL. A total of 1 μL of the amplified product from the first PCR reaction was used as the target DNA in the second reaction. *Hepatozoon* sp. DNA detected in a naturally infected maned-wolf (*Chrysocyon brachyurus*) was used as a positive control [71]. Sterile ultrapure water (Nuclease-Free Water, Promega Corporation) was used as a negative control.

### 4.6. Agarose Gel Electrophoresis

The amplified products were subjected to horizontal electrophoresis on a 1.5% agarose gel stained with ethidium bromide (0.5 µL/mL) in TEB running buffer pH 8.0 (44.58 M Tris-base; 0, 44 M boric acid; 12.49 mM EDTA). Electrophoresis was performed at 100 V/50 mA for 40 minutes. To determine the size of amplified products, a 100 base pair molecular weight marker (Life Technologies^®^) was used. The results were visualized and analyzed using an ultraviolet light transilluminator coupled to a computer image analysis program (ChemiDoc Imaging System, Bio-Rad^®^).

### 4.7. Purification of PCR Amplified Products and Sequencing

After the PCR assays for *Anaplasma* spp., *Ehrlichia* spp., piroplasmids, and *Hepatozoon* spp., the amplified products were purified using Exosap IT PCR Product Cleanup Reagent (Applied Biosystems, Foster City, CA, USA), according to the manufacturer’s recommendations. The sequencing of the amplified products was performed through an automated system based on the method of chain termination by dideoxynucleotide [72]. The sequencing was performed in the ABI PRISM 3700 DNA Analyzer (AppliedBiosystems) sequencer at the Center for Biological Resources and Genomic Biology (CREBIO-FCAV-UNESP).

### 4.8. Phylogenetic Analyses

The sequences obtained were submitted to a quality-screening test using Phred-Phrap software (version 23) [73,74] to evaluate the quality of the electropherograms and to obtain the consensus sequences from the alignment of the sense and antisense sequences. The BLASTn program [75] was used to compare the obtained nucleotide sequences with previously deposited sequences in the GenBank database [76]. The sequences saved in “FASTA” format were aligned with other homologous sequences of each agent retrieved from the database (GenBank), using the Mafft software [77] and edited via Bioedit v. 7.0.5.3 [78]. The “best of it” evolutionary model was selected from the sequence alignment matrices using the jModelTest 2 software [79]. W-IQ-Tree software was used for the choice of the evolutionary model following BIC criterion and for phylogenetic analysis by the maximum likelihood method (available online: http://iqtree.cibiv.univie.ac.at/) [80], while clade support indices were evaluated through bootstrap analyses [81] of 1000 repetitions. The editing of phylogenetic trees as well as rooting (via outer group) were performed using the Treegraph 2.0.56-381 beta software [82].

## 5. Conclusions

The present study showed the occurrence of TBA (*E. canis, Anaplasma* sp., *B. vogeli*, and *Cytauxzoon*) in cats from southeastern and northern Brazil, expanding the list and geographical distribution of such hemoparasites in cats. ‘*Candidatus* Anaplasma amazonensis’, recently detected in sloths from northern Brazil, was described for the first time in cats. The dog-associated *B. vogeli*, *E. canis,* and *A. platys* were molecularly detected in cats from the present study, suggesting environmental infestation with *R. sanguineus* sensu lato, the vector for all three hemoparasites. This is the first report of piroplasmids infecting cats in northern Brazil. Coinfection by *Cytauxzoon* and other TBA (*Ehrlichia, Anaplasma*, and *Babesia/Theileria*) was reported in the present study, which raises the need for veterinary practitioners’ awareness of cats parasitized by multiple TBA.

## Figures and Tables

**Table 1:** Co-positivity by tick-borne agents in cats sampled in southeastern (states of São Paulo e Minas Gerais) and northern (state of Rondônia) Brazil.

	Co-Positivity			Positivity for Only One Agent			
State	*Cytauxzoon* + *Anaplasma*	*Cytauxzoon* + *Ehrlichia*	*Cytauxzoon* + *Babesia*/ *Theileria*	*Anaplasma*	*Ehrlichia*	*Babesia*/ *Theileria*	*Cytauxzoon*
São Paulo	2	0	0	8	1	1	35
Minas Gerais	6	1	1	13	6	0	57
Rondônia	0	0	0	0	0	1	4
**Total**	8	1	1	21	7	2	96

**Table 2:** Percentage of identity assessed by BLASTn of *Anaplasma* sp., *Babesia* sp. and *Cytauxzoon* sp. sequences detected in cats from Brazil.

Cat ID	Target Gene	Query Length (bp)	Query-Coverage (%)	E-Value	Identity (%)	GenBank Acession Numbers
MG03	*rrs*	281	100	4 × 10^−^^143^	100	‘*Candidatus* Anaplasma amazonensis’—*Bradypus variegatus* from Brazil (MT199833)
MG21	*rrs*	372	100	0	100	‘*Candidatus* Anaplasma amazonensis’—*Bradypus variegatus* from Brazil (MT199831)
MG31	*rrs*	543	100	0	97.61	*Anaplasma phagocytophilum*—*Hydropotes inermis* from Korea (KR611598)
MG33	*rrs*	398	99	0	99.75	*Anaplasma* spp.—*Nasua nasua* from Brazil (MT019560)
SP79	*rrs*	522	99	0	100	*Anaplasma* sp.—*Mazama gouazoubira* from Brazil (JF952891)
ROI01	18S rRNA	766	100	0	100	*Babesia vogeli*—cat from China (MN067709)
MG68	18S rRNA	763	100	0	100	*Babesia vogeli*—cat from China (MN067709)
MG71	18S rRNA	218	100	3 × 10^−108^	100	*Cytauxzoon* sp.—*Leopardus pardalis* from Brazil (MT458054)
MG52	18S rRNA	248	99	3 × 10^−^^124^	100	Cytauxzoon sp.—*Leopardus pardalis* from Brazil (MT458054)
MG125	18S rRNA	118	100	6 × 10^−^^53^	100	*Cytauxzoonsp.—Leopardus pardalis* from Brazil (MT458054)
SP121	18S rRNA	140	100	5 × 10^−^^65^	100	*Cytauxzoonsp.—Leopardus pardalis* from Brazil (MT458054)
SP131	18S rRNA	140	100	5 × 10^−^^65^	100	*Cytauxzoonsp.—Leopardus pardalis* from Brazil (MT458054)
ROI06C	18S rRNA	273	100	1 × 10^−^^138^	100	*Cytauxzoonsp.—Leopardus pardalis* from Brazil (MT458054)

**Table 3:** Description of primers, amplicons size and thermal sequences used in conventional and nested PCR assays for *Ehrlichia, Anaplasma, Babesia/Theileria, Cytauxzoon* and *Hepatozoon*.

Agents	Primer Sequences	Size (bp)	Thermal Sequences	References
*Anaplasma* spp. (*rrs* gene)—Screening External primers - gE3a - gE10R Internal primers - gE2 - gE9f	5′-CACATGCAAGTCGAACGGATTATTC-3′ 5′-TTCCGTTAAGAAGGATCTAATCTCC-3′ 5′-GGCAGTATTAAAAGCAGCTCCAGG-3′ 5′-AACGGATTATTCTTTATAGCTTGCT-3′	932 546	94 °C for 5 min 40 cycles: 94 °C for 30 sec, 55 °C for 30 sec and 72 °C for 1 min 72 °C for 5 min	[52]
*Anaplasma spp.* (ITS—23S-5S) - *ITS2F* - ITS2R	5′-AGGATCTGACTCTAGTAACGAG-3′ 5′-CTCCCATGTCTTAAGACAAAG-3′	300	94 °C for 2 min, 35 cycles; 94 °C for 30 sec, 58 °C for 30 sec, 72 °C for 1 min 72 °C for 5 min.	[54]
*Anaplasma* spp. (*gltA* gene) External primers - F4b - Rb1 Internal primers - EHR-CS136F - EHR-778R	5′-CCGGGTTTTATGTCTACTGC-3′ 5′-CGATGACCAAAACCCAT-3′ 5′-TTYATGTCYACTGCTGCKTG-3′ 5′-GCNCCMCCATGMGCTGG-3′	800 600	95 °C for 5 min, 40 cycles: 95 °C for 30 sec, 55 °C for 30 sec and 72 °C for 1 min 72 °C for 5 min.	[53]
*Ehrlichia* spp. (*dsb* gene)—Screening - dsb-330 (F) - dsb-728 (R)	5′-GATGATGTCTGAAGATATGAAACAAAT-3′ 5′-CTGCTCGTCTATTTTACTTCTTAAAGT-3′	409	95 °C for 2 min; 50 cycles: 95 °C for 15 sec, 58 °C for 30 sec and 72 °C for 30 sec 72 °C for 5 min	[55]
*Anaplasma* spp. /*Ehrlichia spp.* (*groEL* gene) - groEL124-F1 - groEL808-R1	5′-ATTAAGCCAGAAGAACCATTAGC-3′ 5′-TACTGCAATATCACCAAGCATATC-3′	680	95 °C for 5 min, 40 cycles: 95 °C for 30 sec, 54 °C for 30 sec and 72 °C for 30 sec 72 °C for 5 min	[56]
*Ehrlichia* spp. *(sodB* gene) - *sodbEhr1600-F* - *sodbEhrl600-R*	5′-ATGTTTACTTTACCTGAACTTCCATATC-3′ 5′-ATCTTTGAGCTGCAAAATCCCAATT-3′	600	94 °C for 3 min; 55 cycles: 94 °C for 10 sec; 58 °C for 10 sec; 72 °C for 15 sec 72 °C for 30 sec 72 °C for 5 min	[57]
*Ehrlichia* spp. (*omp-1* gene) External primers - *conP28-F1* - *conP28-R1* Internal primers - *conP28-F2* - *conP29-R2*	5′-AT(C/T)AGT(G/C)AAA(A/G)TA(T/C)(A/G)T(G/A)CCAA-3′ 5′-TTA(G/A)AA(A/G)G(C/T)AAA(C/T)CT(T/G)CCTCC-3′ 5′-CAATGG(A/G)(T/A)GG(T/C)CC(A/C)AGA(AG)TAG-3′ 5′-TTCC(T/C)TG(A/G)TA(A/G)G(A/C)AA(T/G)TTTAGG-3′	300	94 °C for 3 min, 35 cycles: 94 °C for 1 min, 50 °C for 1 min and 72 °C for 2 min 72 °C for 5 min	[58]
Piroplasmida (18S rRNA)- Screening External primers - BTF1 - BTR1 Internal primers - BTF2 - BTR2	5′-GGCTCATTACAACAGTTATAG-3′ 5′-CCCAAAGACTTTGATTTCTCTC-3′ 5′-CCGTGCTAATTGTAGGGCTAATAC-3′ 5′-GGACTACGACGGTATCTGATCG-3′	800	94 °C for 3 min, 58 °C for 1 min, 72 °C for 2 min 45 cycles: 94 °C for 30 sec, 58 °C for 20 sec and 72 °C por 30 sec 72 °C por 7 min Annealing temperature of the 2nd reaction = 62 °C	[60]
*Babesia* sp./*Theileria* sp. (*cox-1* gene) External primers - Bab_for1 - Bab_Rev1 Internal primers - Bab_for2 - Bab_rev2	5′-ATWGGATTYTATATGAGTAT-3′ 5′-ATAATCWGGWATYCTCCTTGG-3′ 5′-TCTCTWCATGGWTTAATTATGATAT-3′ 5′-TAGCTCCAATTGAHARWACAAAGTG-3′	924	95 °C for 1 min, 35 cycles; 95 °C for 15 sec, 45 °C for 30 sec and 72 °C for 1 min 72 °C por 10 min Annealing temperature of the 2nd reaction = 49 °C	[61]
*Babesia* sp./*Theileria* sp. (*hsp70* gene) - Hsp70F1 - Hsp70R1	5′-CATGAAGCACTGGCCHTTCAA-3′ 5′-GCNCKGCTGATGGTGGTGTTGTA-3′	740	95 °C for 5 min 35 cycles: 95 °C for 15 sec, 60 °C for 30 sec and 72 °C for 30 sec 72 °C for 5 min	[62]
*Babesia* sp./*Theileria* sp. (*β-tubulin* gene) - Tubo3 - Tubo63F	5′-CAAATWGGYGCMAARTTYTGGGA-3′ 5′-TCGTCCATACCTTCWCCSGTRTACCAGTG-3′	600	94 °C for 5 minues 30 cycles: 94 °C for 40 sec, 55 °C for 1 min and 72 °C for 90 sec; 72 °C for 5 min	[63]
*Babesia* sp./*Theileria* sp. (*cytB* gene) External primers - Bc_cytB_F1 - Bc_cytB_R1 Internal primers - Bc_cytB_F2 - Bc_cytB_R2	5′-TGGTCWTGGTATTCWGGAATG-3′ 5′-AAGMYARTCTYCCTAAACATCC-3′ 5′-RATKAGYTAYTGGGGAGC-3′ 5′-GCTGGWATCATWGGTATAC-3′	580	95 °C for 5 min 40 cycles: 95 °C for 45 sec, 55 °C for 45 sec and 72 °C for 45 sec. 72 °C por 10 min; Temp. Annealing temperature of the 2nd reaction = 52 °C	[64]
*Babesia* sp./*Theileria* sp./*Cytauxzoon* sp. (intergenic region ITS-1) External primers - ITS15C - ITS13B Internal primers - ITS15D - ITS13C	5′-CGATCGAGTGATCCGGTGAATTA-3′ 5′-GCTGCGTCCTTCATCGTTGTG-3′ 5′-AAGGAAGGAGAAGTCGTAACAAGG-3′ 5′-TTGTGTGAGCCAAGACATCCA-3′	500	94 °C for 1 min 35 cycles: 94 °C for 30 sec, 52 °C for 30 sec and 72 °C for 1 min. 72 °C for 5 min; Annealing temperature of the 2nd reaction = 49 °C	[65]
*Cytauxzoon* sp. (18S rRNA)—Screening - CytF - CytR	5′-GCGAATCGCATTGCTTTATGCT-3′ 5′-CCAAATGATACTCCGGAAAGAG-3′	300	95 °C for 5 min 40 cycles: 95 °C for 45 sec, 59 °C for 45 sec and 72 °C for 1 min. 72 °C for 5 min	[66]
*Hepatozoon* sp. (18S rRNA)—Screening External Primers - HAM1 - HPF2 Internal primers - 4558 - 2733	5′-GCCAGTAGTCATATGCTTGTC-3′ 5′-GACTTCTCCTTCGTCTAAG-3′ 5′-GCTAATACATGAGCAAAATCTCAA-3′ 5′-CGGAATTAACCAGACAAAT-3′	1120	1st reaction: 95 °C for 3 min 40 cycles: 95 °C for 1 min, 56 °C for 1 min and 72 °C for 1 min. 72 °C for 7 min 2nd reaction: 94 °C for 3 min 40 cycles: 94 °C for 1 min, 55 °C for 2 min and 72 °C for 2 min. 72 °C for 10 min	[67,68,69]

## Data Availability

The obtained nucleotide sequences were deposited in Genbank under the following accession numbers OM069302-OM069306; OM069367-OM069374.
